
JOURNAL INFORMATION
==============================
NLM Title Abbreviation: Inter Econ
Journal ID: Inter Econ
NLM Journal ID: 101086399

Inter Economics

ISSN: 0020-5346

ARTICLE INFORMATION
==============================
PMCID: PMC7276099
PMID: 32536718
DOI: 10.1007/s10272-020-0897-x
Article ID: 897
Article version: 1
Subjects: Letter from America

Coronavirus Pandemic: Europe Is Once Again Forged in a Crisis

Eichengreen Barry eichengr@econ.berkeley.edu

grid.47840.3f 0000 0001 2181 7878 Department of Economics, University of California, Berkeley, 30 Evans Hall, MC #3880, Berkeley, CA 94720-3880 USA
Electronic publication date: 2020 Jun 7
Print publication date: 2020
Volume: 55
Issue: 3
First page: 199
Last page: 200
Copyright: © The Author(s) 2020
License: Open Access This article is licensed under a Creative Commons Attribution 4.0 International License, which permits use, sharing, adaptation, distribution and reproduction in any medium or format, as long as you give appropriate credit to the original author(s) and the source, provide a link to the Creative Commons licence, and indicate if changes were made.
The images or other third party material in this article are included in the article’s Creative Commons licence, unless indicated otherwise in a credit line to the material. If material is not included in the article’s Creative Commons licence and your intended use is not permitted by statutory regulation or exceeds the permitted use, you will need to obtain permission directly from the copyright holder.
To view a copy of this licence, visit http://creativecommons.org/licenses/by/4.0/.
License URL: https://creativecommons.org/licenses/by/4.0/

issue-copyright-statement: © The Author(s) 2020

==============================

To an American, the debate around how the European Union should respond to the COVID-19 crisis has a familiar ring. Europe has been debating debt mutualization, transfer union and fiscal federalism for years. The pandemic is just another opportunity for sounding familiar themes.

But the crisis is also a reminder that there is nothing distinctively European about this rhetoric. Closer to home (my home, anyway, where I am spending considerable time at the moment), we see southern state politicians like Florida Senator Rick Scott impugning northern states like New York for their profligacy. Or, as President Trump put it on Twitter, “Why should the people and taxpayers of America be bailing out poorly run states (like Illinois, as [sic] example) and cities, in all cases Democrat run and managed, when most of the other states are not looking for bailout help?” Northern Europeans have no monopoly on such sentiments. Crises, wherever they occur, have a way of bringing out sectional divisions and reinforcing cultural stereotypes.

Divisions and stereotypes notwithstanding, the need for economic and financial assistance for EU member states with limited fiscal space has never been more pressing. Is there a way forward? At one extreme, one can imagine relying on existing mechanisms. The European Stability Mechanism (ESM), the eurozone’s rescue fund, could loan to the full extent of its capacity. But loans, as opposed to grants, are not what already over-indebted governments need. Moreover, the ESM is set up to loan subject to conditions, and under present circumstances conditionality would be onerous if not downright offensive.

In addition, the European Central Bank (ECB) can do ‘whatever it takes’, to coin a phrase, to support the prices of bonds issued by member states to finance their budget deficits. In the limit, the ECB could resort to yield curve control, pegging interest rates on such bonds in the manner of the Federal Reserve in 1946-51 and the Bank of Japan today. Eventually, however, the ECB would receive pushback from critics objecting that it was straying into the conduct of fiscal policy. In any case, the central bank would only be kicking the can down the road. The fiscal and financial crisis would be back, in other words, as soon as the ECB curtailed its security purchase operations.

At the other end of the spectrum, one can imagine a ‘Great Leap Forward’ involving the significant pooling of fiscal resources, along with transfers to member states in need. One is reminded of Jean Monnet’s observation that Europe is forged in crises; this is certainly the kind of crisis that justifies forging. One is also reminded of how planning in the U.S. and British Treasuries for a new international economic and financial order after World War II was already well advanced during the war – indeed, the Bretton Woods Conference occurred while fighting was still underway.

Like World War II, the coronavirus crisis is a unique historical moment. The middle of the crisis is not too early to start thinking about the post-crisis European order. European leaders, like John Maynard Keynes and Harry Dexter White in the 1940s, need to signal that they recognize the singularity of the moment and that they have plans for capitalizing upon it. Otherwise, public support for the EU will wither, and there is reason to worry about whether the Union itself will survive.

But a significant pooling of existing revenue sources and a greatly expanded EU budget will require political decisions. Inevitably, there will be lengthy negotiations. There may have to be referenda in some member states – in some cases, repeated referenda perhaps, as in Denmark in 1992-93. Anything less would be undemocratic. And abrogating democracy in response to the crisis is not a sustainable way forward. The EU is not Hungary.

There is, however, a middle way. It would start with a modest EU budget targeted at health and testing needs, funded by new rather than existing revenue sources and distributed by an independent fiscal council. A Pandemic Fund is no different, conceptually or politically, from the Structural Funds and the Common Agricultural Policy. A tightly targeted Pandemic Fund could be ramped up without extended politicking or a treaty change. This could be accomplished while at the same time avoiding toxic terms like transfer union and debt mutualization. Assuming that it proves its worth, the Pandemic Fund could eventually become the basis for an expanded EU fiscal capacity.

The case for EU support for member states hit hardest by the pandemic is compelling. Their needs are overwhelming. Moral hazard is not an issue; no one thinks that governments will tolerate more COVID-19 cases in order to get more funding. If the members of the New Hanseatic League will not help other member states that desperately need hospital beds, ventilators, testing and – one hopes – vaccines, then the EU has no future.

Relying on new taxes rather than existing revenue sources, meanwhile, will avoid complaints that national fiscal prerogatives are being arrogated by Brussels. One new source is the proposed tax on internet companies. The problem is that a digital tax would plausibly raise only $1 billion a year. (Some estimates are slightly higher, but no matter.) $1 billion of tax income could be used to pay the interest on $50 billion of newly issued perpetual bonds, but even this would be inadequate. Ramping up testing to the levels required to fully reopen the European economy may cost twice this much by itself.

But one can also imagine other new revenue sources. The pandemic is a reminder that humanity's fate still rests with the natural world. It is just as much a reminder as it is a distraction from the dangers of climate change. When better to start raising petrol taxes than when Europeans are not driving?

Which member states exactly should receive the money? Again, politics threaten to intrude. The solution is for parliaments to first agree on the general criteria governing distribution, and then for the distribution of resources to be delegated to a fiscal council of independent experts. That council would release its decisions and would be subject to audit. This transparency would help to hold it accountable in the court of public opinion. This, of course, is precisely the institutional arrangement that governs the ECB.

Critics will complain about a proliferation of independent technocratic committees. They will observe, as they always do, that fiscal policy is more fundamentally distributive, and therefore political, than monetary policy, the implication being that it cannot be removed from the political sphere.

This objection has two flaws. First, central bank policy, most especially the unconventional credit and financial policies being implemented by central banks at the moment, is no less fundamentally distributive. Second, this idea is not a proposal for delegating fiscal policy to an independent committee of technocrats. Rather, it is a proposal for delegating the distribution of a specific pot of resources – a limited pot initially – and doing so subject to a politically determined mandate.

A political decision can be taken subsequently to close this program down, if and when the pandemic ends, or alternatively to expand it. In the latter case, there will be fiscal and institutional innovations on which to build. Europe will again have been forged in crisis.

Then again, it is easier to recommend a way forward for someone else's economy than your own.

Open Access funding provided by ZBW — Leibniz Information Centre for Economics.

Barry Eichengreen, University of California, Berkeley, USA.

